# Is there any association between prostate-specific antigen screening frequency and uptake of active surveillance in men with low or very low risk prostate cancer?

**DOI:** 10.1186/s12894-019-0502-4

**Published:** 2019-08-05

**Authors:** Kerri Beckmann, Netty Kinsella, Henrik Olsson, Anna Wallerstedt Lantz, Tobias Nordstrom, Markus Aly, Jan Adolfsson, Martin Eklund, Mieke Van Hemelrijck

**Affiliations:** 1 Translational Oncology & Urology Research, Comprehensive Cancer Centre, King’s College London, 3rd Floor, Bermondsey Wing, Guy’s Hospital, London, SE1 9RT UK; 2 University of South Australia, Centre for population Health Research Adelaide Australia, Adelaide, Australia; 3 Department of Urology, The Royal Marsden Hospital, London, UK; 4 Department of Medical Epidemiology and Biostatistics, Karolinska Institutet, Stockholm, Sweden; 5 Department of Molecular Medicine and Surgery, Karolinska Institutet, Stockholm, Sweden; 6 CLINTEC-Department, Karolinska Institutet, Stockholm, Sweden; 7 Unit of Epidemiology, Institute of Environmental Medicine, Karolinska Institutet, Stockholm, Sweden

**Keywords:** Active surveillance, PSA testing, Screening, Prostate biopsy, Prostate neoplasm

## Abstract

**Background:**

Patient-related factors such as concern about cancer are believed to influence both men’s decisions to undergo prostate specific antigen (PSA) testing and to have definitive treatment if diagnosed with low risk prostate cancer (PCa). The potential link between screening frequency and choice of active surveillance (AS) for low risk disease has not been studied previously. Our aim was to investigate whether there is any association between PCa screening frequency or previous negative prostate biopsy and uptake of AS among men with low risk PCa.

**Methods:**

This register-based study included all men ≤75 years from Stockholm who were diagnosed with low risk PCa from 2008 to 2014 (*n* = 4336). Pre-diagnostic PSA testing and biopsy histories were obtained from the Stockholm PSA and Biopsy Register, a population-based register for the Stockholm country. The association between previous screening/biopsy history and AS uptake (based on primary treatment recorded in the National Prostate Cancer Register) was examined using multivariable logistic regression.

**Results:**

Forty seven percent of men with low risk PCa underwent AS. Uptake was associated with older age, very low risk disease, more recent diagnosis and absence of family history. None of the screening/biopsy measures (testing frequency, mean interval, PSA velocity, highest pre-diagnostic PSA or prior negative biopsy) were associated with uptake of AS among men with low risk PCa. Generalisability to settings with different policies and practices may be limited.

**Conclusion:**

We found no evidence that screening frequency and negative biopsy influence uptake of AS among Swedish men with low risk PCa. Further research is required to determine factors that still present barriers for men taking up AS.

**Electronic supplementary material:**

The online version of this article (10.1186/s12894-019-0502-4) contains supplementary material, which is available to authorized users.

## Background

The incidence of prostate cancer (PCa) has been increasing over the past two decades in a large number of countries, primarily due to the availability and uptake of prostate-specific antigen (PSA) testing as a means of early detection [1]. While prostate specific antigen PSA testing has considerable value in clinical follow-up after diagnosis, its utility with respect to detecting PCa is contentious^,^ [2]. Most professional society guidelines do not recommend population-based PSA screening, due to increased risk of overdiagnosis and overtreatment, but do support shared decision making between physicians and men who are concerned about PCa risk [3].

Increased availability and use of PSA testing as a screening tool has led to an increase in the incidence of low and intermediate risk disease as well as stage migration in PCa across a number of countries [4], with a large proportion of PSA-detected cancers classified as low-grade/low-volume indolent disease, with little metastatic potential [5]. Many clinical practice guidelines now recommend active surveillance (AS) for low risk PCa, in an attempt to reduce the unnecessary morbidity of overtreatment [6]. Even so, variation in the uptake of AS occurs between and within countries [7, 8].

Recent reviews have identified several factors that appear to drive the uptake of AS [9–12]. These include patient related factors, family and social supports, clinician factors, health systems and health policy [9]. Concern about cancer progression and control of cancer are commonly mentioned as factors that impact choice to undertake AS [12, 13]. Similar factors have been reported to drive PSA screening, where men identify concerns about cancer, the need for peace of mind and control over one’s health as reasons for undergoing PSA testing [14, 15]. Hence, the choice to undergo PSA testing, and to have definitive treatment if diagnosed with PCa, may be correlated through a common concern to optimize health outcomes. On the other hand, men with prior experience of prostate biopsy may be deterred from choosing AS due to negative adverse side effects following biopsy [16, 17], or because they have less confidence in the accuracy of biopsy procedures to detect cancer progression [18]. Several interview and internet content-based studies have suggested the psychological burden associated with ongoing monitoring during active surveillance, as well as the morbidity associated with repeat biopsy, may be linked to reduced uptake AS [19]. However, other studies have emphasized the process of repeated monitoring while on active surveillance as reassuring [20].

To our knowledge, the question of whether frequency of PSA testing or a negative prostate biopsy before diagnosis influence the uptake of AS has not previously been addressed. The primary aim of this study was to determine whether frequency of pre-diagnostic PSA testing was associated with uptake of AS among men diagnosed with low or very low risk PCa. Our secondary aims were to determine whether the experience of a negative prostate biopsy or changes in PSA levels prior to diagnosis influenced the likelihood of choosing AS. The quantitative findings of this work will add to the qualitative studies investigating patient and physician behaviour, so that combined they can help us develop better educational and supportive tools for patients and healthcare professionals.

## Methods

To assess the relationship between PSA screening history and uptake of AS we utilized data from the Stockholm PSA and Biopsy Register, a population-based register which contains information on every PSA test and prostate biopsy for men residing in Stockholm County since 2003. PSA test results and biopsy reports were obtained from the three official laboratories within the Stockholm region (Karolinska University Laboratory, Aleris and Unilabs) providing near complete population coverage of all PSA and biopsy tests undertaken in the county since the start of the register [21]. All PSA and biopsy data were provided electronically, directly from laboratory records to the register. Data were also collected on demographic characteristics, all PCa diagnoses, history of PCa among first degree male relatives and hospitalisations (since 1998), through linkages with established national registries using the unique Swedish personal identification number. A previous quality study of the National Prostate Cancer Register (NPCR), which was the source of cancer related information, indicates coverage of 98% of PCa cases compared with the National Cancer Register, and on average 90% completeness of clinical variables collected [22].

In Sweden there is no formal PCa screening program. However, non-symptomatic men can request a “screening” test from their general physician. Hence data on PSA tests in this study include a mixture of tests relating to symptoms and testing based on requests from patients. However, they pertain only to PSA tests undertaken before diagnosis. In addition, treatment choices in Sweden are usually made through shared decision-making between patient and treating clinician.

Our study cohort consisted of all men residing in the Stockholm County between 2008 and 2014 who were diagnosed with low risk PCa, defined as PSA < 10 ng/ml, Gleason score < =6, and clinical stage T1–2. Men aged over 75 years at diagnosis were excluded. This cohort was chosen to represent men with 10 years life expectancy who would have been eligible for AS according to Swedish guidelines [23].

The main outcome of interest was uptake of AS. For our main analyses we derived the measure of AS uptake from the NPCR [24] based on primary treatment reported to the register 6 months post-diagnosis. When treatment data were missing (*n* = 485, 11%) men were considered not to have undergone AS. For sensitivity analyses, we reclassified AS based on linkage with the inpatient register to identify men who had undergone radical prostatectomy or radiotherapy as inpatients within 6 months of diagnosis. This resulted in reclassification of four men from the AS group to active treatment and 476 men with unknown treatment to the AS group. This reclassification assumes that men were on AS if treatment data were missing in NPCR and no active treatment was identified in the inpatient register.

Measures of pre-diagnosis PSA and biopsy history were derived from the Stockholm PSA and Biopsy Register. These included: total number of PSA tests; total number of PSA tests excluding any likely repeat tests for abnormal/inconclusive results (i.e. tests within 60 days of the previous PSA test); highest total PSA; PSA velocity (defined as change in total PSA/year from first test to PSA at diagnosis); and number of prior negative biopsies. Biopsies performed within 60 days of diagnosis were considered part of diagnostic workup and were excluded. For the main analysis, all measures were derived for the five-year period before PCa diagnosis to ensure equivalence with respect to period of observation.

Data on potential confounders included: age and year of diagnosis; Charlson comorbidity index (CCI) [25], civil status (married, not married); education level (<high school, high school, post-secondary and university); family history of PCa (any first-degree family member had PCa); symptoms at presentation (yes/no); and risk category (e.g. very low versus low risk). In accordance with Swedish guidelines, very low risk PCa was defined as those with T1c disease, ≤4 positive cores (of ≥8 cores) and PSA density (total PSA/prostate volume) < 0.15 μl/cm^3^. Most men would have been assessed through 8–10 core TRUS biopsy prior to treatment decision. Use of MRI was not specified in the Swedish guidelines during the study period and thus has not been considered as a contributing factor in this study.

Multivariable logistic regression models were undertaken to determine the relationship between number of previous PSA tests and uptake of AS in the eligible cohort, with simultaneous adjustment for all confounders and prior number of negative biopsies. Separate models were also conducted for each of the specific PSA measures, and adjusted odds ratio and 95% confidence intervals are reported. Further subgroup analyses were undertaken according to risk category (very low, low risk) and period of diagnosis (2008–2010, 2011–2012, 2013–2014). Finally, we undertook the following sensitivity analyses to assess impact of potential errors in outcome and exposure measures: 1) analyses using all PSA and biopsy data from 2003 to include all available data, 2) analyses excluding men who did not have primary treatment recorded in NPCR, and 3) analysis with treatment reassigned according to linkage with the inpatient register.

Ethics approval was obtained from the Stockholm Regional Ethics Committee.

## Results

Of the 4336 eligible participants, 2038 (47%) had AS recorded as the primary treatment for PCa. Table 1 shows the cohort characteristics according to whether participants underwent AS. Those who choose AS were slightly older, presented less frequently with symptoms, and had lower volume disease (e.g. fewer positive cores, lower maximum core length, and higher percent with very low risk disease). There was a strong trend toward more men taking up AS in the later years, especially 2013–2014.

**Table 1:** Cohort characteristics

Characteristics *N* = 4336	Active surveillance		*p*-value
	no	yes	
Total - n (%)	2298 (53)	2038 (47)	
Age - mean (SD)	62.2 (6.6)	63.9 (5.7)	< 0.001
Cancer characteristics- median (IQR)			
PSA at diagnosis	5 (3.8–6.6)	4.6 (3.4–6.1)	< 0.001
Max cancer length	5 (2–11)	2 (1–4)	< 0.001
Positive cores	2 (1–4)	1 (1–2)	< 0.001
Prostate volume	35 (26–46)	40 (30–51)	< 0.001
Stage:			
T1c - n (%)	1800 (78)	1827 (90)	< 0.001
T2 - n (%)	498 (22)	211 (10)	
Risk Category (Swedish guidelines) - n (%):			
Very low risk	552 (24)	1114 (55)	< 0.001
Low risk	1746 (76)	924 (45)	
Reason for presentation - n (%)			
Health check	1412 (61)	1542 (76)	< 0.001
LUTS	397 (17)	297 (15)	
Other symptoms	257 (11)	153 (8)	
Unknown	218 (10)	41 (2)	
Comorbidity (CCI at diagnosis)			
0	2012 (88)	1764 (86)	0.531
1	179 (8)	178 (9)	
2	75 (3)	72 (4)	
3+	30 (1)	32 (2)	
Year of diagnosis - n (%)			
2008	337 (15)	127 (6)	< 0.001
2009	413 (18)	160 (8)	
2010	323 (14)	154 (8)	
2011	242 (11)	178 (9)	
2012	247 (11)	209 (10)	
2013	313 (14)	415 (20)	
2014	423 (18)	795 (39)	
Education level - n (%)			
< High school	323 (14)	320 (16)	0.073
High school	940 (41)	867 (43)	
Post high school< 3 yrs	330 (14)	296 (15)	
University	687 (30)	541 (27)	
missing	18 (1)	14 (1)	
Civil Status			
Not married/widowed	749 (33)	719 (35)	0.063
Married/partner	1549 (67)	1319 (65)	
History of PCa in family - n (%)			
No	1713 (75)	1621 (80)	< 0.001
Yes	583 (25)	415 (20)	
TREATMENTS - n (%)			
Active surveillance	0 (0)	2038 (100)	
Curative	1604 (69)	0 (0)	
Watchful waiting	209 (9)	0 (0)	
Treatment unknown	485 (21)	0 (0)	

PSA testing and prostate biopsy history are shown in Table 2 according to AS status. The two treatment groups were similar with respect to mean number of previous PSA tests, maximum total PSA, minimum free to total PSA ratio, and number of previous prostate biopsies. The mean PSA velocity was slightly lower for men who underwent AS.

**Table 2:** PSA testing and prostate biopsy history in the 5 years prior to diagnosis of prostate cancer

Measures	Active surveillance		*p*-value
	no	yes	
Prior PSA tests - *Mean (sd)*			
Total no. PSA tests	4.0 (2.6)	3.9 (2.7)	0.322
No. PSA tests excluding confirmatory tests	3.3 (2.3)	3.2 (2.2)	0.597
Screening interval (days)^a^	304 (281)	333 (313)	0.003
Mean pre-diagnostic total PSA level	4.6 (1.9)	4.3 (2.1)	0.001
Highest pre-diagnostic total PSA level	5.8 (3.1)	5.4 (3.6)	0.001
PSA velocity (ng/mL/year)^a^	0.46 (1.6)	0.42 (1.7)	0.001
No. biopsies prior to diagnosis *No. (%)*			
None	2001 (87)	1771 (87)	0.934
1	235 (10)	211 (10)	
2	48 (2)	46 (2)	
3+	14 (1)	10 (0.5)	

^a^among men with 2 or more tests during previous 5 years

Results of multivariable logistic regression analysis examining the effect of frequency of PSA tests and prior number of prostate biopsies on uptake of AS are shown in Table 3. Uptake of AS was more likely among older age groups (odds ratio [OR]: 1.69; 95% confidence interval [CI]: 1.37–2.20, 65-69 yrs. versus 55-59 yrs) and those with very low risk PCa (OR: 2.95; 95%CI: 2.49–3.40) and less likely among men with a family history of PCa (OR: 0.82; 95%CI: 0.69–0.97) and men who were married (OR: 0.80; 95%CI: 0.67–0.93). The odds of AS uptake also increased with more recent years of diagnosis. No association was observed with respect to either number of PSA tests or prostate biopsies during the 5 years before diagnosis.

**Table 3:** Factors associated with uptake of up active surveillance, among men with low and very low risk prostate cancer in Stockholm Sweden 2008–2014

Factors	crude			adjusted^a^		
	OR	95% CI	*p*-value	OR	95% CI	*p*-value
Age group						
< 55 yrs	0.64	0.49–0.84	< 0.001	0.61	0.46–0.81	< 0.001
55-59 yrs	1.00	ref	–	1.00	ref	–
60-64 yrs	1.14	0.90–1.29	0.170	1.19	0.96–1.49	0.117
65-69 yrs	1.80	1.50–2.15	< 0.001	1.69	1.37–2.10	< 0.001
70-74 yrs	1.37	0.74–0.98	0.005	1.70	1.30–2.21	< 0.001
Civil status						
Not married	1.00	ref	–	1.00	ref	
Married	0.89	0.78–1.01	0.063	0.80	0.67–0.93	0.004
Education level						
Less than high school	1.07	0.87–1.32	0.436	1.06	0.86–1.31	0.565
Completed High school	1.00	ref	–	1.00	ref	–
3 yrs. post high school	0.97	0.81–1.17	0.376	0.96	0.78–1.20	0.738
University	0.85	0.73–0.99	0.019	0.87	0.73–1.03	0.104
Family history of PCa						
No	1.00	ref	–	1.00	ref	–
Yes	0.75	0.65–0.87	< 0.001	0.82	0.69–0.97	0.023
CCI						
0	1.00	ref	–	1.00	ref	–
1	1.15	0.92–1.43	0.235	0.98	0.76–1.26	0.785
2+	1.17	0.88–1.55	0.567	1.05	0.76–1.47	0.785
Presented with symptoms/LUTS						
no	1.00	ref	–	1.00	ref	–
yes	0.63	0.54–0.72	< 0.001	0.88	0.74–1.03	0.113
Prostate volume	1.01	1.01–1.02	< 0.001	1.00	1.00–1.01	0.197
Risk category						
Low	1.00	ref	–	1.00	ref	
Very low	3.81	3.35–4.34	< 0.001	2.95	2.49–3.40	< 0.001
Diagnosis year (cont. 2008–2014)	1.34	1.30–1.38	< 0.001	1.35	1.30–1.40	< 0.001
No. PSA tests in past 5 yrs (cont.)	0.75	0.65–0.87	0.322	0.99	0.97–1.02	0.672
No. negative biopsies in past 5 yrs (cont.)	1.01	0.88–1.14	0.947	1.11	0.94–1.30	0.214

*CCI* Charlson comorbidity index, *CI* confidence interval, *LUTS* lower urinary tract symptoms, *OR* odds ratio, *PCa* prostate cancer, *PSA* prostate specific antigen, *ref.* reference

^a^simultaneously adjusted for all factors

Results for associations between specific PSA/biopsy measures and uptake of AS are shown in Table 4. There was no association between uptake of AS and pre-diagnostic total PSA concentration or PSA velocity prior to diagnosis. Odds were slightly elevated for any prior negative biopsy (OR: 1.11, 95%CI: 0.90–1.38), though the difference was not statistically significant. Results were similar when considering low and very low risk categories separately. Similarly, analyses for different diagnostic periods showed no association for PSA screening or biopsy history in any period. (Table 5).

**Table 4:** Adjusted ORs for different measure of PSA testing/results associated with uptake of AS in men with low/very low risk PCa (History for the 5-year period before diagnosis)

PSA and Biopsy measures	OR	95% CI	*p*-value
Total number of PSA tests (continuous)	0.99	0.96–1.02	0.672
Total number of PSA tests excluding repeat tests (test within 60 days of previous test)	0.99	0.96–1.03	0.597
Highest pre-diagnostic total-PSA level (per ng/mL)	0.98	0.95–1.02	0.332
Mean PSA velocity (earliest to diagnostic PSA level)	0.98	0.96–1.01	0.133
Any previous negative prostate biopsy	1.11	0.90–1.38	0.329
Total number of previous biopsies (continuous)	1.04	0.88–1.21	0.669

*CI* confidence interval, *OR* odds ratio, *PSA* prostate specific antigen

ORs derived from separate multivariable logistic regression models adjusted for age, education level, civil status, CCI, family history, symptoms, diagnosis year and risk category (and number of previous PSA tests/biopsies)

**Table 5:** Adjusted ORs for uptake of AS in relation to previous number of PSA tests and any prior negative biopsy, stratified by 1) risk and 2) period of diagnosis (History for the 5-year period before diagnosis)

Stratified by:	Number of PSA tests			Number of biopsies		
	OR	95% CI	*p*-value	OR	95% CI	*p*-value
Risk category						
Very low risk (*n* = 1568)	0.99	0.96–1.04	0.681	1.06	0.78–1.38	0.702
Low risk (*n* = 2292)	0.99	0.96–1.03	0.984	1.14	0.94–1.38	0.187
Year of diagnosis						
2008–2010 (n = 1,339)	0.98	0.94–1.03	0.485	1.21	0.95–1.56	0.121
2011–2012 (*n* = 818)	1.02	0.98–1.07	0.366	0.93	0.71–1.21	0.594
2013–2014 (*n* = 1703)	0.99	0.93–1.05	0.791	1.00	0.73–1.37	0.995

*CI* confidence interval, *OR* odds ratio, *PSA* prostate specific antigen

ORs derived from separate multivariable logistic regression models adjusted for age, education level, civil status, CCI, family history, symptoms, diagnosis year and risk category (simultaneous adjustment for no. PSA tests and biopsy)

### Sensitivity analyses

Results were essentially unchanged from our main findings for all PSA test and biopsy measures in each of the sensitivity analyses undertaken. These included when AS status was reclassified based in hospital admission data, when cases with unknown treatment were excluded, and when PSA and biopsy history included all available data from 2003 rather than the 5-year period before diagnosis. (Additional file 1: Table S1).

## Discussion

Our study is the first to explore whether frequency of PSA testing, PSA results or the experience of a negative prostate biopsy influence uptake of AS among men diagnosed with low risk PCa. We found no association between uptake of AS and frequency of PSA testing, PSA velocity or highest total PSA concentration prior to diagnosis. Uptake of AS was also not associated with a history of previous negative biopsy. Our findings applied equally to men with low or very low risk disease and across time periods. Equivalent results from our sensitivity analysis investigating the influence of missing data indicate robust findings despite some missing treatment information.

Uptake of AS was higher among older men, those with very low risk disease and those more recently diagnosed, while it was lower among those with a family history of PCa. These findings are encouraging as they indicate increased uptake over time and among those most likely to be over-treated. Increasing trends toward AS among men with low risk PCa have been reported in a number of large cohorts, though considerable variations remains between countries, practices and physicians [7]. Current estimates of AS uptake range from 74% in Sweden in 2014 (Swedish National Prostate Cancer Register [8]), 40% in USA in 2010–13 (Cancer of the Prostate Strategic Urologic Research Endeavor [26]), 40% in Australia in 2012 (Victorian Prostate Cancer Registry [27]) to 21% in Canada in 2010 (Ontario Cancer Registry [28]). The rate of uptake in our study (47%) is within the range of contemporary cohorts worldwide. However, the relatively high rate of uptake in Sweden may limit the generalizability of our findings to other countries.

Our finding of no association between AS uptake and frequency of PSA testing does not support the hypothesis that screening frequency and choice of treatment are linked through patient health-seeking behaviours, at least not for men residing in Stockholm. While anxiety and cancer worry have been suggested as potential factors influencing frequency of PSA testing, as well as choice of PCa treatment [12, 13], evidence for their effect on screening and treatment decisions is tenuous. Quantitative studies examining the relationship between anxiety and PSA screening have yielded mixed results, including positive [29] and negative [30] associations with higher anxiety, a U-shaped relationship [31] and no association [14]. Similarly, fear of disease progression and wanting control over cancer are commonly mentioned reasons for not choosing AS in several qualitative studies [13], but there is little quantitative evidence demonstrating the influence of such psychological factors on uptake of AS.

Several recent studies and reviews have highlighted the importance of more systemic factors on uptake of AS, such as physician’s recommendation [11], and local or national policies and practices [8, 9, 11, 19]. For example, clinician bias for active treatment can reduce uptake of AS [9, 10, 12], while good patient-physician relationships, shared decision-making, a multidisciplinary approach and specific policies supporting AS facilitate greater uptake of AS [9, 10]. In the current study, AS uptake increased sharply in the later years. The strong endorsement of AS for low risk PCa in the 2013 update of Swedish guidelines is likely to explain this trend, lending support for the argument that clinician recommendation and the health policy environment have a major influence on uptake of AS [8]. Lack of data on treating clinician precluded direct assessment of clinician variability in the current study.

In addition, we found no evidence to support the hypothesis that having had a prior negative biopsy influences the decision to undergo AS. Nor did we find any evidence that higher serum PSA levels or higher PSA velocity prior to diagnosis, which may increase patients’ concerns about the potential for disease progression, influenced the uptake of AS in men diagnosed with low risk disease. We had expected that having had a previous biopsy would be negatively associated with uptake of AS due to some men having experienced pain, discomfort or side effects, such as hematuria, infection and sepsis, which can affect up to 1 in 3 men undergoing biopsy [16, 17]. Equally, uptake of AS might be reduced due to a lack of confidence in biopsy procedures if it was perceived that cancer had been missed at an earlier biopsy [18]. Our findings do not indicate a negative association between prior history of biopsy and AS uptake.

Diagnostic and surveillance pathways for men with PCa cancer are rapidly evolving, particularly with respect to the use of MRI to detect significant cancer and to monitor disease progression [32]. Further research is required to determine whether this provides men with increased reassurance and confidence to commence AS.

The strengths of the study are its large size and the population-based nature of the data with accurate linkage resulting in complete coverage of all PSA testing and biopsy procedures undertaken in the Stockholm County since 2003. While some inaccuracy in exposure measures is possible given a small proportion of men may have undergone testing in another County during the study period, the effects are likely to be negligible. Primary treatment data were extracted from national registries for Stockholm residents so that treatment outside the County but within Sweden would be captured. Hence, our findings of no association are unlikely to be due to lack of power or misclassification of exposure, which would bias toward null findings. Furthermore, a substantial proportion of men in the study population had no treatment record at 6 months. However, sensitivity analyses using all available data and various adjusted measures of AS yielded consistent results. Since our study did not include assessment of any psychological measures (e.g. anxiety or cancer concern) we cannot draw direct conclusions that such factors are not influential in decision making around screening or treatment. The lack of data on treating clinician is a further limitation, given the potential influence physicians have on patients’ treatment decisions. Finally, generalisability of these findings outside of Sweden may be limited, given the rates of AS uptake are relatively high in Sweden and that PSA testing and AS are likely to be driven by health care system-specific factors as well as the local policy environment (i.e. clinical guidelines) [9].

## Conclusion

We found no evidence that pre-diagnostic PSA screening frequency or negative biopsies influence uptake of AS among Swedish men with low risk PCa. Given uptake of AS is not yet 100%, further research is required to determine what factors still present barriers for men taking up AS.

## Additional file

Additional file 1: **Table S1.** Sensitivity analyses. Adjusted ORs for different measures of PSA/biopsy history associated with uptake of AS in men with low/very low risk PCa. (DOCX 15 kb)

## Data Availability

The datasets used for analyses in this study are not publicly available as they are register based data, but are available from martin.eklund@ki.se on reasonable request.
