# Supplementary material for: Is there any association between prostate-specific antigen screening frequency and uptake of active surveillance in men with low or very low risk prostate cancer?
Source: BMC Urol. 2019 Aug 5;19:73. doi: 10.1186/s12894-019-0502-4 (PMC6683376; doi:10.1186/s12894-019-0502-4)
Supplement: Supplementary file 1 — Table S1. Sensitivity analyses. Adjusted ORs for different measures of PSA/biopsy history associated with uptake of AS in men with low/very low risk PCa. (DOCX 15 kb) [file 12894_2019_502_MOESM1_ESM.docx]

**Additional file 1**

**Table S1: Sensitivity analyses**

**Adjusted ORs for different measures of PSA/biopsy history associated with uptake of AS in men with low/very low risk PCa**

| **PSA and Biopsy measures** | **OR** | **95% CI** | **p-value** |
| --- | --- | --- | --- |
|  |  |  |  |
| ***History of all PSA tests / biopsies from 2003:*** |  |  |  |
| Total number of PSA tests (continuous) | 0.99 | 0.97-1.01 | 0.221 |
| Total number of PSA tests excluding repeat tests | 0.99 | 0.97-1.01 | 0.399 |
| Highest pre-diagnostic total-PSA level (per ng/ml) | 0.99 | 0.98-1.02 | 0.386 |
| Mean PSA velocity (earliest to diagnostic PSA level) | 0.98 | 0.96-1.01 | 0.124 |
| Any previous negative prostate biopsy | 1.14 | 0.92-1.41 | 0.242 |
| Total number of previous biopsies (continuous) | 1.05 | 0.90-1.23 | 0.534 |
|  |  |  |  |
| ***Excluding cases with no treatment assigned in NPCR:*** |  |  |  |
| Total number of PSA tests (continuous) | 0.98 | 0.95-1.01 | 0.176 |
| Total number of PSA tests excluding repeat tests | 0.97 | 0.94-1.01 | 0.106 |
| Highest pre-diagnostic total-PSA level (per ng/ml) | 0.99 | 0.96-1.01 | 0.252 |
| Mean PSA velocity (earliest to diagnostic PSA level) | 0.99 | 0.97-1.01 | 0.167 |
| Any previous negative prostate biopsy | 1.17 | 0.93-1.48 | 0.172 |
| Total number of previous biopsies (continuous) | 1.06 | 0.89-1.24 | 0.513 |
|  |  |  |  |
| ***Assignment of active surveillance based on linkage with hospital separation data:*** |  |  |  |
| Total number of PSA tests (continuous) | 0.98 | 0.95-1.01 | 0.195 |
| Total number of PSA tests excluding repeat tests | 0.97 | 0.94-1.01 | 0.137 |
| Highest pre-diagnostic total-PSA level (per ng/ml) | 0.98 | 0.96-1.01 | 0.140 |
| Mean PSA velocity (earliest to diagnostic PSA level) | 0.99 | 0.97-1.01 | 0.198 |
| Any previous negative prostate biopsy | 1.13 | 0.89-1.41 | 0.266 |
| Total number of previous biopsies | 1.09 | 0.92-1.27 | 0.318 |
|  |  |  |  |

CI: confidence interval; OR: odds ratio; PSA: prostate specific antigen

ORs derived from separate multivariable logistic regression models adjusted for age, education level, civil status, CCI, family history, symptoms, diagnosis year and risk category (and number of previous PSA tests/biopsies).
